# Organ wide toxicological assessment of common edible herbs and their mixtures as used in home remedies

**DOI:** 10.5620/eaht.2023012

**Published:** 2023-06-29

**Authors:** Chigeru Wodi, Ebere Ezaka, Boniface N. Ukwah, Ukpai A. Eze

**Affiliations:** 1 Department of medical laboratory science, Ebonyi State University, Abakaliki, Nigeria; 2 Leicester School of Allied Health Sciences, De Montfort University, Leicester, United Kingdom

**Keywords:** medicinal plants, home remedies, herbal formulation, organ toxicity

## Abstract

The use of home remedies for medicinal purposes, most of which are edible plants has continued to be a practice in many homes. However, there has been an increasing report of chronic use with lethal effect. Among the commonly used herbal/ medicinal plants were ginger, garlic and lemon. These were seen to be prevalent across continents with brewing and crude extraction being the most means of consumption. This study investigated the organ wide toxicity of this extract following chronic consumption of crude extract. Twenty-five albino Wister rats, five in each group were used for this experiment. Each animal received 0.5ml/kg body weight of either ginger extract, garlic extract, lemon juice, or a mixture of equal volumes of all three extract (v/v) respectively twice daily for seven (7) days. Statistics were represented as ±SE; P≤0.05 was considered significant. Previous studies have shown that moderate consumption of these medicinal plants were beneficial and have shown no deleterious effect. This study observed no change in the weight of the experimental animals. The weight of the animals continued to increase except for the group that received lemon and the mixture, but these were not significant. It was observed that chronic consumption induced organ wide toxicity to include the liver, kidney, intestinal epithelium, stomach, and pancreas. These were shown to alter tissue architecture and the cell morphology. Packed cell volume was reduced in the lemon and the group that received a combination of all extracts (p=o.03). Blood differentials showed changes in levels. An elevated basophil level was observed in ginger and garlic (p<0.0001; p=0.0006). Monocyte levels increased progressively across each group when compared to the control with the most elevated level seen in the group that received the mixture (p<0.0001). Lymphocyte count was reduced across all the groups that received the extract except for animals that received ginger. This study suggests the application of caution among users of these medicinal plants and continues to draw attention to the need for harmonization and standardization of safe use doses.

## Introduction

The importance of plants cannot be over emphasized. Apart from providing the nutritional needs for mammals including humans, there has been increasing advocacy in recent times for the need to explore and maximize the medicinal properties of these plants. Medicinal plants continue to provide the raw materials for new drugs [1]. It is estimated that over 1300 medicinal plants are used in Europe [2] and in the United States over 75% of prescribed drugs are plant based [1]. The developed world is seeing an exponential rise in demand for complementary and alternative medicine, especially in aromatherapy [3, 4, 5]. In developing countries, 80% of habitats are completely dependent on herbal drugs to meet their healthcare needs with 25% of prescribed drugs sourced from wild plants [6]. Globally, the demand for herbal drugs, natural health products of secondary products of medical plants is at its peak [7, 2]. However, the toxicity of these herbal drugs is largely unexplored, especially in low to middle-income countries.

Several factors have contributed to this increased demand for complementary and herbal medicine, and the reasons can be seen to differ from country to country. In the developing countries, the likely reason for use of complementary medicine would be lack of time to see a physician, poor hospital infrastructure in rural and some urban communities, fear of possible misdiagnosis especially where patients have non-specific symptoms or general malaise and lastly, freedom of choice with referral from friends and family suggestive of choice [8, 5]. Whereas patients’ discomfort in discussing their health condition especially where confidentiality of health information is a concern, influence of religion and spiritual consciousness [9, 5], cost of modern medicine and lack of accessibility to healthcare facilities and personnel are the likely reasons for herbal and complementary medicine use in developed countries.

Although drug discovery from edible and medicinal plants continues to provide an important alternative for developing countries, this is not without numerous challenges. Among the huddles encountered in the adoption of edible medicinal plants is the problem of dosage and toxicity. This is the case with edible medicinal plants which are consumed daily as part of food and treatment of ailments and hence, with the prejudice of causing no harm or toxicity even with overdose [10]. Apart from the intrinsic toxicity associated with misuse and overdose, contamination from pesticides, heavy metals, mycotoxins, and hygiene and method of processing poses external risk factors for toxicity [11, 10]. Some examples which are of key interest to the research is the increased consumption of crude extracts of ginger (*Zingiber officinale*), garlic (*Allium sativum*) and lemon (*Citrus limon*) extract during the peak periods of the covid-19 pandemic [12, 13]. These edible plants have been shown to have valuable pharmaceutical, cosmetic and culinary (healthy food) properties [12,14] and are so called superfood. The reliability and increased consumption of ginger, garlic and lemon were based on both traditional and documented evidence of their anti-inflammatory, antipyretic and relief of symptoms of cold and flu and respiratory disorders [14, 15, 16, 17, 18].

Even though the medicinal properties of these edible plants have been documented, there are conflicting reports with regards to toxicity and classification of toxicity due to lack of scientific standard and objective experimental data. There is no adequate data about toxicity of targeted organs, safe dose range and safe window of minimum effective dose. Therefore, this research explored the toxic effect of each edible herbal extract as well as their mixtures as used in home remedies.

## Materials and Methods

### Sample collection, Identification and Preparation (Crude extraction)

Bulbs of ginger (*Zingiber officinale*), garlic (*Allium sativum*) and lemon (*Citrus limon*) fruits were purchased from the local market and authenticated in the Department of Plant Science, Ebonyi State University, Abakaliki, Nigeria. A total of 600g each of fresh bulbs of ginger and garlic previously described were grounded and suspended in a litre of sterile distilled water (w/v) respectively. These were stored at room temperature for 4 hours after which they were sterile filtered. The lemon fruit was washed, cut into two parts and the juice squeezed out. The juice was sterile filtered and diluted with sterile distilled water at 300ml per Litre (v/v) of sterile distilled water. A mixture of equal volumes of Garlic extract, Ginger extract and lemon juice was also prepared. All crude extracts were refrigerated at 4°C throughout the period of administration to slow fermentation. The extracts were normalized to room temperature before each administration.

### Test Animals

A total of twenty-five (25) Albino Wister rats were used. The animals weighed between 200–260g. Animals were maintained and housed in cages under standard environmental conditions (25°C±3°C, 12-hour light/ dark cycle) and acclimatized for two weeks before being used for the experiment. Food and water were given ad libitum throughout the experiment.

### Study Design

After acclimatization, the Wister rats were randomly divided into five groups with five animals in each group. The groups and the crude extract received are outlined in Table 1. Animals were weighed daily in the last seven days of the acclimatization and their mean weight documented. Once administration of the extract was started, experimental rats were weighed each day before administration of the extract. Both weights were compared.

Animals in each category (A, B, and C) received 0.5ml/kg body weight of either ginger extract, garlic extract, or lemon juice, respectively twice daily for seven (7) days. Group D was treated with 0.5ml/kg body weight of a mixture of an equal volume of garlic extract, ginger extract, and lemon juice twice daily for seven (7) days. Crude extract was administered orally using oral gavage. The control Group E was given a corresponding volume of distilled water throughout the experiment. All procedures used in this study conformed to the guidelines set down for research involving animals [19]. All experimental procedures were approved by the Animal Ethical Committee of the Faculty of Health Sciences, Ebonyi State.

### Collection of Blood Sample

Three millilitres of blood sample was collected from the abdominal aorta using syringes and was dispensed directly into a commercially prepared Ethylene Ethylenediaminetetraacetic acid (EDTA) container. The samples were analysed using a haematology autoanalyzer (Mindray B12 right med biosystem, India).

### Sacrifice of Animals and Histological Procedure

At the end of the experimental period, the experimental Wister rats were grossly observed for general physical characteristics and sacrificed under anaesthesia using chloroform (Sigma-Aldrich, Germany). The animals were euthanised using 5 ppm of chloroform inhalation exposures for 3 minutes and sacrificed immediately for tissue collection. This exposure did not cause any organ toxicity in control animals without herbal treatment (Fig 3A, 4A, 5A, 6A, 7A). The extent of organspecific damage by chloroform exposure is dependent on the rates of production of its toxic metabolites by cytochrome P450 2E1 enzyme [21]. Therefore, the very low dose as used in this study is not expected to induce organ toxicity and also, the inhalation exposure duration used is not enough for the metabolic conversion of chloroform to occur. It has been demonstrated that the inhalation exposures of female B6C3F1 mice to 10 ppm or less of chloroform did not significantly induce organ damage, even when exposure periods were up to 18 h per day [22]. A midline incision was made on the anterior abdominal wall and the organs of interest isolated and fixed in 10% formal saline (Sigma-Aldrich, Germany) for routine histological processing. Tissue processing of specimens from the test animals involved fixation in 10% formal saline, dehydration in ascending concentration of ethyl alcohol (Sigma-Aldrich, Germany) and cleared in xylene (Fisher Scientific, South Africa). Tissues were impregnated with molten paraffin (Fisher Scientific, South Africa), embedded in paraffin wax, and tissue sections were cut using microtome. The prepared sections were brought to water and stained using Haematoxylin and Eosin (H&E) (Sigma-Aldrich, Germany) and allowed to dry. The dried sections were mounted using DPX (Morrison chemicals, Nigeria) and examined microscopically.

### Statistical analysis

The values presented in graphs are mean ± standard error of mean (Mean ± SEM) for treatments and control groups calculated from triplicate determinations (n = 3). Data were analysed with GraphPad PRISM software version 9.1 (San Diego, CA). The average weight of the animals during the period of acclimatization was compared to their average weight at the end of treatment. Differences between treated and control groups were analysed by one-way ANOVA followed by Dunnett's procedure for multiple comparisons. Differences were reported as statistically significant at p ≤ 0.05. Significant effects are represented on graphs by p ≤ 0.05 (*), p ≤ 0.01 (**), p ≤ 0.001 (***) and p ≤ 0.0001 (****).

## Results

**Figure 1.**
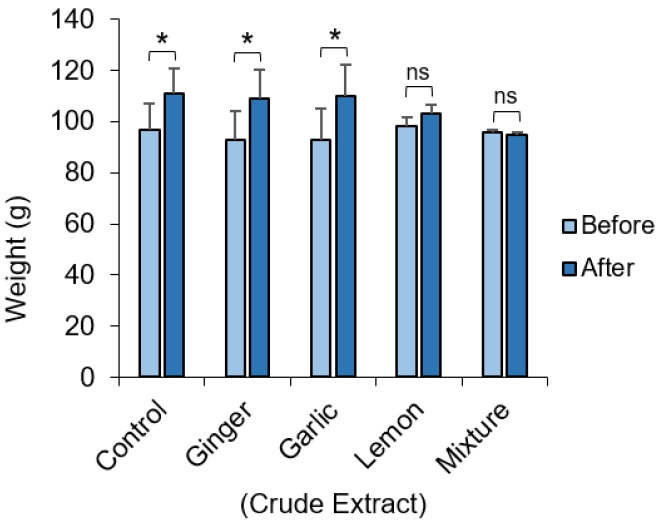
Difference in the weight of experimental animals. Albino Wister rat before and after administration of crude extract of ginger, garlic and lemon and a mixture of all extract. The average weight of animals in each group before and after administration of the crude extract. Data represents mean ± S.E. *p ≤ 0.05, one-way ANOVA (n=3)

The weight of the animals before administration of the crude extract was compared to the weight obtained for the same set of animals after administration of the crude extract (Figure 1). Average weights of the animals receiving the crude extract of ginger and garlic were unaffected and they continued to gain significant weight (p=0.02). Weight gain in the group receiving the lemon appeared to be slowed and was not significantly different to the pre-administration weight. Experimental animals in groups receiving an equal mixture of all extract showed apparent decrease in weight, although this was not statistically significant.

### Crude extract of ginger, garlic and lemon have varying effect on blood cells

**Figure 2.**
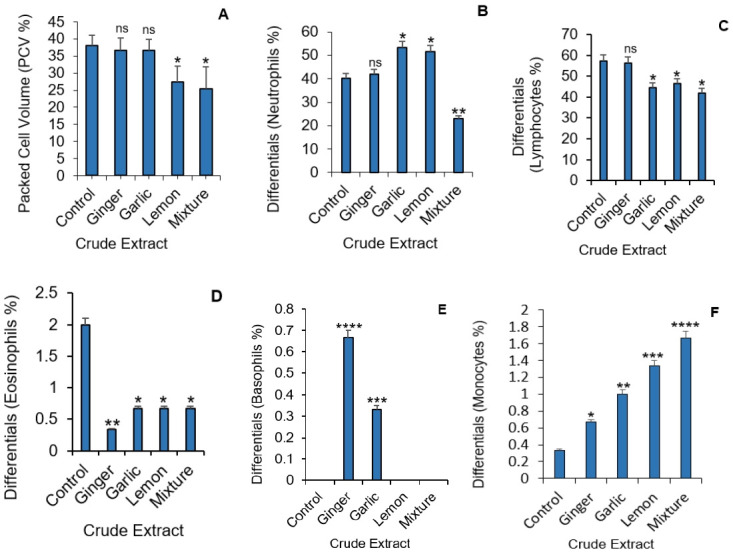
Effect of crude extract on packed cell volume (PCV) and white blood cell differentials (%) in Albino Wister rat. (A) Average PCV level for each treatment group. Differentials for Neutrophils for each group (B); Lymphocyte for each group (C); levels of Eosinophils (D); percentage of Basophil (E) and Monocyte count (F). Data represents mean ± S.E. *p ≤ 0.05, **p<0.01, ***p<0.001 and ****p<0.0001. one-way ANOVA (n=3).

We observed first-hand the effect of all three-crude extract on blood cells and to see if there was a trend. Results were compared to the control since animals were kept under the same condition rather than standards as several factors can affect the blood parameters. The packed cell volume (PCV) also known as the haematocrit test, was performed to see if 0.5ml/kg of crude extracts of the ginger, garlic and lemon will have any effect on levels of red blood cells. Only lemon and combination of all three crude extracts resulted in a reduction in PCV levels (Figure 2A).

In addition, differentials for white blood cell (WBC) count shows variable effects for each extract on the individual differentials. The combination of all three crude extracts appear to significantly decrease neutrophil count when compared to the control (Figure 2B). Whereas neutrophil levels in groups treated with ginger were unaffected, groups treated with garlic and lemon extract showed significant increase in neutrophil levels.

Garlic, lemon, and a combination of all extracts significantly decreased lymphocyte count when compared to the control with the group receiving ginger extract unaffected (Figure 2C). Eosinophil count was found to be reduced in all groups when compared to the control, with the ginger group having the least count (Figure 2D). Interestingly, ginger and garlic were found to significantly increase circulating levels of basophils (Figure 2E). There was a progressive increase in monocyte level observed across the groups. The group that received ginger showed the least level of circulating monocyte while the group that received a combination of all extracts showed significantly higher levels of circulating monocytes (Figure 2F).

### Wide toxicity of crude extract of ginger, garlic and lemon on organ tissues

Further analysis was performed to evaluate the toxic effects of the crude extract of ginger, garlic and lemon on organ tissues. Tissue morphology of the liver, kidney, stomach, small intestine and pancreas were observed on H&E stained thin histological sections. These organs were selected as they are either in direct contact with the extract following consumption or involved in its metabolism.

#### Toxicity of crude extract on liver cells

**Figure 3.**
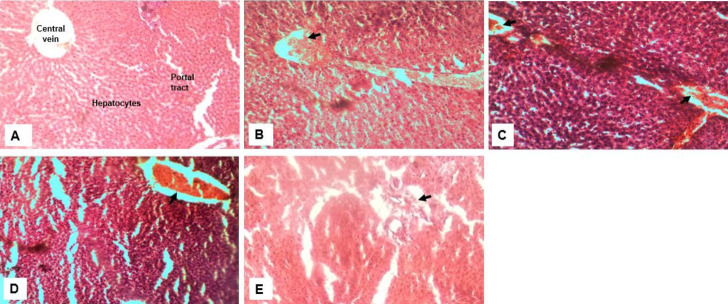
Effect of crude extract on the histology of the liver. Representative image (A) Control -Normal liver morphology showing the central vein, portal tract and hepatocytes radiating from the central vein; (B) Liver from group that received ginger; (C) Received crude garlic extract; (D) Group that received Lemon juice and (E) equal mixture of all extract - sinusoidal congestion (arrows) and hyperchromasia in the nuclei of the hepatocytes. (H&E, magnification ×400).

Cells of the liver showed extensive distortion in tissue architecture (Figure 3) with the most distortion observed in the group that received a combination of all extracts (Figure 3E) and the least distortion seen in the group receiving ginger (Figure 3B). All groups showed hyperchromasia (Figure 3B-E) with the garlic and lemon group most intensely stained (Figure 3C&D). Sinusoidal congestion was also observed in all groups except the control (Figure 3A) and widening of the portal tract which was pronounced in the group receiving lemon and a combination of all crude extract.

#### Toxicity of crude extract on the kidney

**Figure 4.**
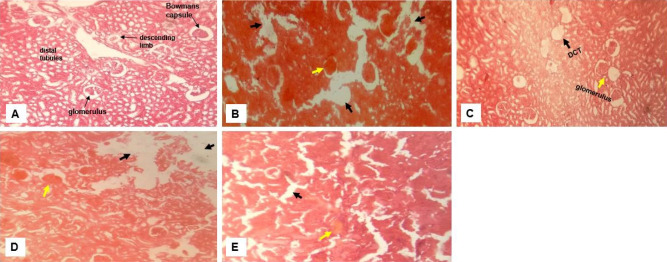
Effect of crude extract on the histology of the Kidney. Representative images -Normal kidney morphology showing the Bowmans capsule and glomerular tuft, descending limb of the loop of Henle and distal tubules (A); Kidney from group that received ginger showing distortion of the renal architecture and dilated tubules (long arrows) (B) ; (C) Crude garlic extract with shrunken glomerulus and tubular damage ; (D) Lemon juice with severe glomerular shrinkage and congestion, and tubular damage and (E) Equal mixture of all extracts with severe damage to the renal architecture. (DCT - distal convoluted tubules; tubular damage - black arrow; glomerular shrinkage and congestion - yellow arrows). (H&E, magnification ×400).

Kidney tissues from all group showed varying degrees of distortion to the tissue morphology with the least toxicity observed in the group receiving garlic (Figure 4C). Consistent in all groups is the shrinkage of the glomerulus and dilation of the tubules (Figure 4B-E). Tubular and glomerular congestion were also observed in group given ginger and a mixture of all the extracts (Figure 4B&E).

#### Toxicity of crude extract on the stomach

**Figure 5.**
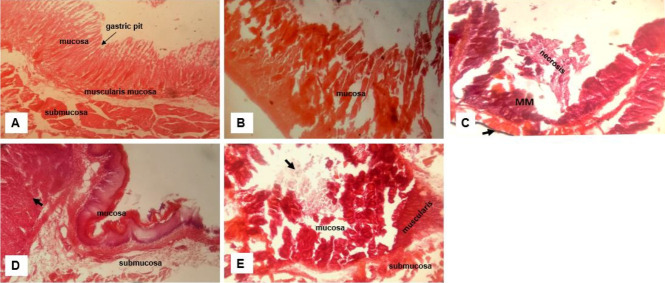
Effect of crude extract on the histology of the Stomach. Representative images - (A) Normal stomach morphology with intact gastric epithelium; (B) Ginger group with eroded epithelium ; (C) Received crude Garlic extract with severe epithelial damage and necrosis and haemorrhagic areas (arrow) and damaged muscularis mucosa (MM) ; (D) Lemon juice group with erosion of the epithelium (arrow) and extended damage to the submucosa and (E) Mixture of all extracts with damaged epithelium, necrosis (arrow) and damage to the muscularis and submucosa. (H&E, magnification ×400).

Stomach epithelia appear to be completely eroded from toxicity of the crude extract. Extensive damages was observed which extended to the submucosa (Figure 5C-E). There were areas of haemorrhagic and necrotic lesions seen on the tissue (Figure 5C-E).

#### Toxicity of crude extract on the small intestine

**Figure 6.**
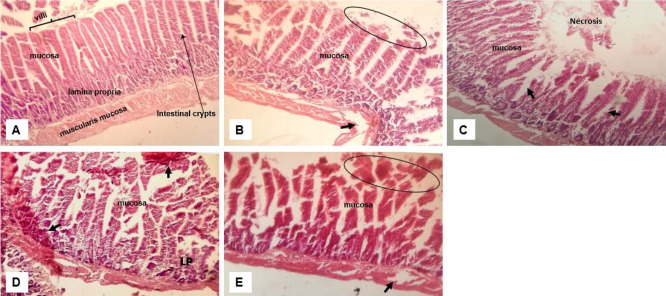
Effect of crude extract on the histology of the small intestine. Normal intestinal architecture; (B) moderate distortion of the mucosa with eroded villi (oval); (C) Garlic extract group with damaged villi with area of necrosis and dilated crypts (arrow); (D) Lemon juice group with erosion of the epithelium and extended to the lamina propria (LP) with haemorrhagic areas (arrows) and (E) Mixture of all extracts with damaged mucosa and necrotic areas (oval) and to the muscularis mucosa (arrow). (H&E, magnification ×400).

Examination of the histology of the small intestine also revealed moderate to severe distortion of the intestinal epithelium (Figure 6). The degree of distortion and toxicity varied with the most damage found in the group that received the mixture which extends to the muscularis mucosa (Figure 6E). Consistent in all groups receiving crude extract are focal lesions, necrosis and dilated crypts. Among all three extracts, apart from the combination, the group that received lemon (Figure 6D) showed more toxicity with lesions extending to the lamina propria and having haemorrhagic spots.

#### Toxicity of crude extract on the pancreas

**Figure 7.**
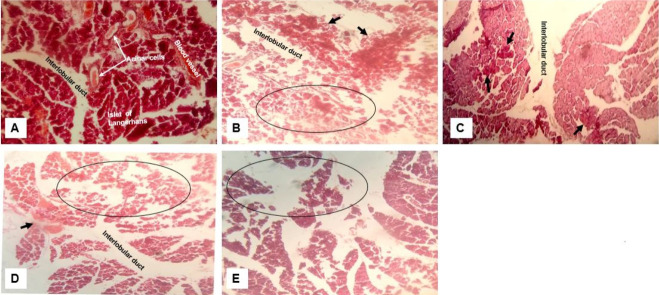
Effect of crude extract on the histology of the pancreas. Normal pancreatic morphology; (B) loss of acini cells (oval) with aggregation of macrophages (arrows) and dilated duct; (C) Garlic group – macrophage infiltration (arrow) and dilated duct; (D) Lemon group - Damaged acini and islet cells (oval) and thickening of blood vessel (arrow); and (E) Mixture of extracts - damaged lobular ducts, islet, and acini cells with inflammation (oval). (H&E, magnification ×400).

The pancreatic tissue sections from each experimental group also showed different degrees of toxicity resulting in the damage of pancreatic acini and islet cells. Interlobular ducts were found to be dilated in all groups (Figure 7B-E). Aggregation of macrophage due to inflammation was observed in groups that received ginger, garlic and lemon (Figure 7B-D). There was also an indication that crude lemon extract was associated with thickening of blood vessels (Figure 7D).

## Discussion

For decades, natural and edible herbs have been used to treat several diseases and in recent times, has continued to provide the raw materials for pharmaceutical companies. With the global health concern that has arisen during the Covid19 pandemic, the dependence on superfood and natural herbs has surged. One study has identified ginger, garlic and lemon among the most consumed food-medicine during the pandemic [12]. These were consumed as teas and spices, and for their known properties in the management of flu, anti-inflammatory and immunomodulatory characteristics [23,24]. However, there has been less focus on the indiscriminate consumption of these herbal medicines and the associated health consequences. Even though it may be assumed the benefits outweigh the risks.

In our study, it was observed that the average weight of the rats given crude extract of ginger and garlic were not significantly affected and they continued to gain significant weight (Fig. 1). This can be attributed to the hypocholesterolemic and hypolipidemic potential of ginger which do not have any significant effect on serum lipoproteins (a) [Lp (a)] levels after supplementation with garlic both in rats and humans [25,26]. We also observed that after 7 days of lemon treatment, the weights of rats increased compared to their weight at baseline, although this was not statistically significant. Also, rats fed with an equal mixture of garlic, ginger and lemon extract showed apparent decrease in weight, although this was not statistically significant (Fig. 1). This would be the synergic effect of the combination of all three extracts, considering that these extracts on their own, except for the lemon, did not induce a significant weight loss in the experimental animals. A previous study reported that albino Wistar rats given ginger-, garlic-, and lemon-based herbal mixtures had the lowest weight gain trend when compared with the positive control [27]. Interestingly, this study shows that fresh juice of garlic-, ginger-, and lemon-based herbal mixtures have low fat residues and therefore, it is not expected to cause significant increase in calories of the fed rats. This further supports the lack of significant effect on weight gain seen in our study.

It was observed that ginger and garlic had no effects on the packed cell volume of exposed animals whereas lemon and the ternary mixture significantly reduced Packed cell volume (Fig. 2). High doses of the herbal extracts appear to suppress lymphocyte function across all treatments when compared to the control. Low levels of eosinophil patocytes were common presentations after exposure to the herbal extracts (Fig. 3). Minimal toxicity to the liver hepatocytes were observed in rats treated with ginger. The hepato-protective effects of ginger have been attributed to its antioxidant activity which inhibits the membranous lipid peroxidation from free radicals that usually cause cell damage and necrosis [34,35]. Treatment with garlic and lemon caused prominent damage to the liver cells in the rats used in this study and an extended synergic effect seen in the group given the mixture. Morphological changes in the liver ultrastructure in animals exposed to garlic extract with associated focal non-specific injury of the hepatocytes is associated to significant reduction in endogenous antioxidants. The sulfoxides present in the garlic extract are thought to be responsible for its toxicity as it readily reacts with –SH groups of enzymes and proteins in the body inhibiting their activity [36, 37]. The action of sulfoxides also alters aspartate aminotransferases (AST) and alanine transaminase (ALT) level. The reverse effect was observed with lemon on liver enzymes (AST, ALT, ALP) and Bilirubin when compared to the control [38, 39], further associating the sulfoxides present in garlic to hepatic toxicity.

Corresponding histological and ultrastructure changes were also found in the kidney across all treatment (Fig. 4). This is no surprise considering the homeostatic and regulatory function of the kidney for micronutrients. We observed that there was shrinkage of the glomerulus and dilation of the tubules with the least distortion to renal tissue morphology observed in the group receiving garlic. Changes in the kidney histology and ultrastructure has also been reported with administration of 1000mg/kg/day of garlic [36] which is consistent with the result of this study. Erosion of the stomach wall and intestinal villus and damage up to the mucosa was reported at the experimental dose of 0.5ml/kg of crude single and ternary mixtures of ginger, garlic and lemon extracts (Fig. 5 & 6). An earlier study has observed taller and wider villus at increased doses from 0.25% to 0.5% of ginger and garlic powder in birds [40]. This suggests that increased doses or concentrations of both extracts can induce excessive cell proliferation and hypertrophy capable of inducing cell damage. Degeneration of the intestinal mucosa, shortening of the intestinal villi, and damage to the intestinal brush border are some of the histological changes reported with use of diet supplemented with heated garlic [41].

We also demonstrated that exposure to ginger, garlic and lemon caused damage to pancreatic acini and islet cells (Fig. 7). In addition, interlobular ducts were found to be dilated in all treated groups. The Insulinotropic effect of ginger and garlic through a direct pancreatic mechanism, if overstimulated as a result of increased dosing may explain and link the toxicity observed in this study to the administered extracts. Combination of ginger and garlic doubled this effect [42] and explains the extensive damage observed with the combination of all three extracts.

The treatment of the Wistar rats with ternary mixtures containing garlic, ginger and lemon produced the most haematological and organ wide toxicity (Fig. 3-7). Herbs consist of several phytochemicals which provide many health benefits when taken in low doses, but their chronic consumption or overconsumption could result in synergistic interaction leading to toxicological effects [43,44, 45]. Several toxic effects of herbal formulations, including carcinogenic, neurotoxic, genotoxic, teratogenic, cytotoxic, nephrotoxic, hepatotoxic, and gastrointestinal toxic effects have been reported [46]. The herbs used in this study are well known as “health foods” and their herbal concoctions are traditionally used in many parts of the world for dietary purposes or the treatment and management of various disease conditions such as diabetes, haemorrhage, inflammatory disorders, obesity, hypercholesterolemia, hypertension, and other cardiovascular disorders [47,48,49].The toxicological effects of these herbs are known to occur when taken at high doses whereas their health promoting benefits are observed at lower doses [46]. Therefore, it is pertinent to highlight the toxic effects of these herbs in conjunction with their health benefits as this would warrant the identification of their safety profiles and reduce the chronic consumption of these herbs, especially in concoction mixtures.

## Conclusions

The results of this study therefore draw attention to the possible multiple organ toxicity of indiscriminate and continuous consumption of ginger, garlic, and lemon formulations and their mixtures. The synergistic haematological and organ-wide toxicity of the ternary mixtures of garlic, ginger and lemon on the treated Wistar rats found in this study warrant more careful toxicological assays to investigate safer consumption doses of such herbal formulations. Although these herbs used in the study are also well known as “health foods” and are used for medical purposes in various parts of the world., it has become pertinent to highlight the toxic effects of the herbs in conjunction with their health benefits as this would warrant the identification of their safety profiles and reduce the chronic consumption of these herbs, especially in pregnant women and infants as this poses serious health risks in this population.

## Figures and Tables

**Table 1.** Groups and the treatment received.

Groups	Treatment Received
A	Ginger (*Zingiber officinale*)
B	Garlic (*Allium sativum*)
C	Lemon (*Citrus limon*)
D	Equal mixture of Garlic, Ginger and Lemon
E	Distilled water

## List of Abbreviations

AST: Aspartate Aminotransferase
ALP: Alkaline Phosphatase
ALT: Alanine Transaminase
DCT: Distal Convoluted Tubules
DPX: Dibutylphthalate Polysturene Xylene
H&E: Haematoxylin and Eosin
IBS: irritable bowel syndrome
LP: Lamina propria
MM: Muscularis mucosa
PCV: Packed Cell Volume
V/V: Volume per Volume
WBC: White Blood Cell
W/V: Weight per Volume
